# 224. Clinical Relevance of Antibiotic Tolerance in Recurrent Gram-negative Bloodstream Infections

**DOI:** 10.1093/ofid/ofad500.297

**Published:** 2023-11-27

**Authors:** Joshua B Parsons, Ashelyn E Sidders, Amanda Z Velez, Blake M Hanson, Felicia Ruffin, Sarah Rowe-Conlon, Cesar A Arias, Vance G Fowler, Joshua T Thaden, Brian P Conlon

**Affiliations:** Duke University Medical Center, Durham, North Carolina; University of North Carolina, Chapel Hill, North Carolina; University of North Carolina, Chapel Hill, North Carolina; The University of Texas Health Science Center, Houston, Texas; Duke University Medical Center, Durham, North Carolina; University of North Carolina, Chapel Hill, North Carolina; Houston Methodist and Weill Cornell Medical College, Houston, TX; Duke University Medical Center, Durham, North Carolina; Duke University School of Medicine, Durham, North Carolina; University of North Carolina, Chapel Hill, North Carolina

## Abstract

**Background:**

Gram-negative bacterial bloodstream infections (GNB-BSI) are common and frequently lethal. Many patients experience recurrent GNB-BSI for unclear reasons. This study explores how antibiotic tolerance may lead to infection relapse.

**Figure 1:**
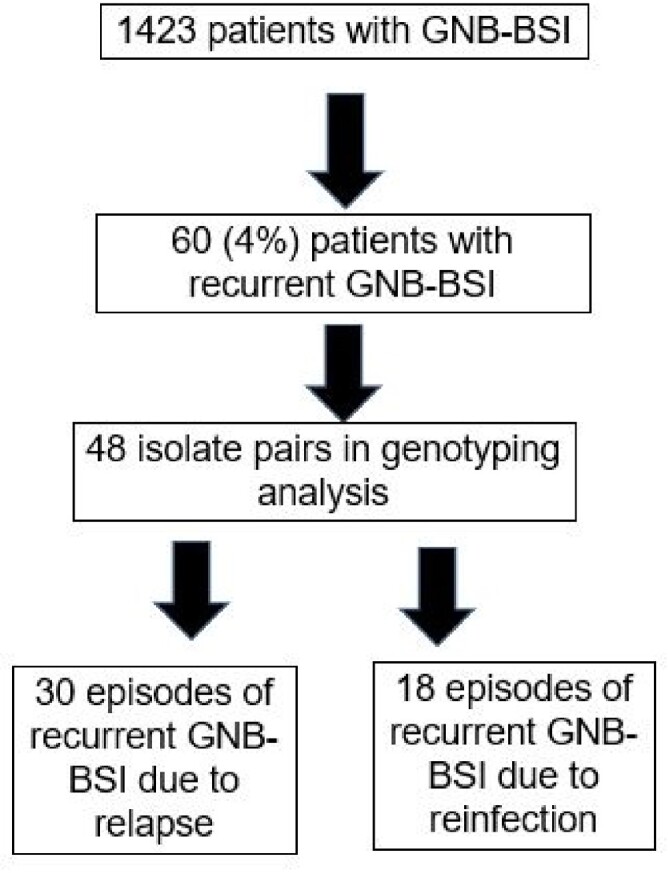
Flow diagram of relapsed versus recurrent infection.

Approximately 63% of recurrent GNB-BSI are due to relapse (almost genetically identical isolate) and 37% due to reinfection (genetically distinct isolate).

**Figure 2:**
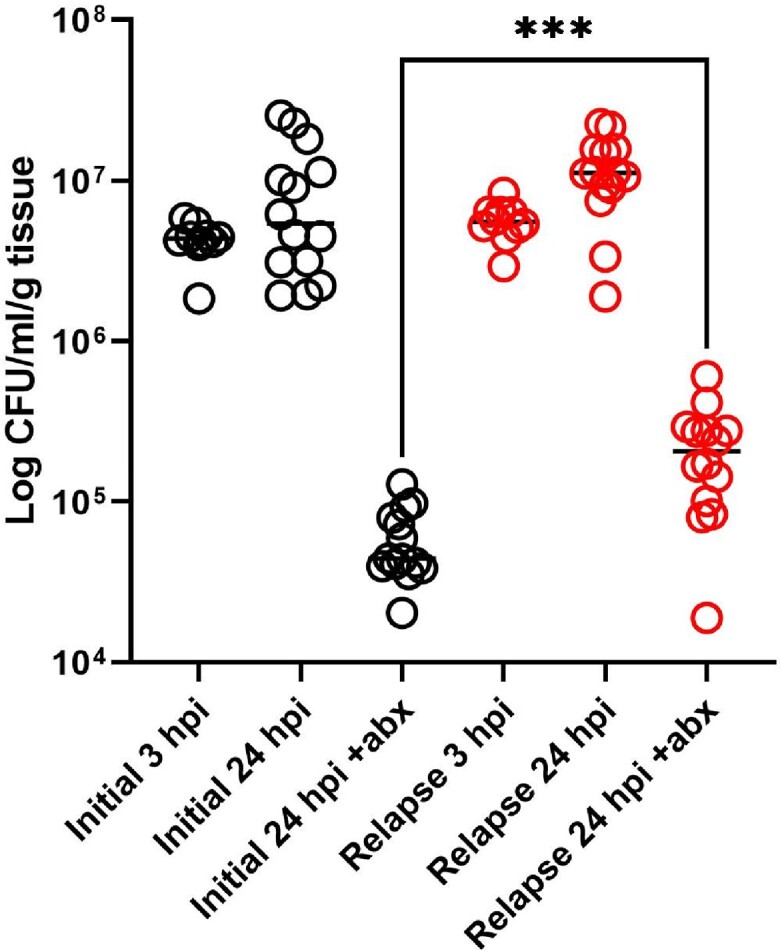
Relapsed GNB-BSI isolate exhibits increased antibiotic tolerance in murine bacteremia model.

BALB/c mice infected with paired initial or relapsed GNB-BSI isolate. 50 mg/kg ertapenem added at 3 hours post infection (hpi). Graph shows recovery of viable bacteria from liver tissue.

**Methods:**

We used a prospective cohort of patients with GNB-BSI at Duke Hospital to identify patients with >1 episode of GNB-BSI due to the same bacterial species. We used whole genome sequencing (WGS) to distinguish reinfection (recurrent infection with different isolate) from relapse (recurrent infection with near-identical isolate). Time-kill curves with meropenem were used to determine the development of antibiotic tolerance between the initial and relapsed *Escherichia coli* isolates. The biological relevance of antibiotic tolerance was tested using a murine bacteremia model.

**Results:**

We determined that 63% (30/48) of recurrent GNB-BSI episodes were due to relapse and 37% (18/48) were due to reinfection. We screened 10 relapsed *E. coli* pairs (initial and relapsed isolates) using meropenem to identify increases in antibiotic tolerance. One isolate pair showed a 100-fold increase in multidrug antibiotic tolerance. We determined the decreased antibiotic killing was due to a loss-of-function mutation in the *ptsI* gene encoding Enzyme I of the phosphoenolpyruvate phosphotransferase system. To test if the *in-vitro* tolerance phenotype translated to decreased antibiotic efficacy *in-vivo*, we developed a murine model of *E. coli* bacteremia. In our murine bacteremia model, the *ptsI* mutant was equally virulent as the wild-type, but exhibited 10-fold less killing during antibiotic treatment.

**Conclusion:**

Our work provides a unique insight into the molecular changes occurring during GNB-BSI and how the pathogen adapts to the host through acquisition of antibiotic tolerance. We address the controversy regarding the clinical relevance of antibiotic tolerance *in-vivo* by providing compelling data that not only do these mutations arise during bloodstream infection in humans, but the presence of antibiotic tolerance *in-vitro* likely leads to decreased antibiotic efficacy *in-vivo*. Further work is required to determine if early detection of antibiotic tolerance could lead to alterations in medical management and ultimately improve patient outcomes.

**Disclosures:**

**Vance G. Fowler, MD, MHS**, Amphliphi Biosciences, Integrated Biotherapeutics; C3J, Armata, Valanbio; Akagera, Aridis, Roche, Astra Zeneca: Advisor/Consultant|Genentech, Regeneron, Deep Blue, Basilea, Janssen;: Grant/Research Support|Infectious Diseases Society of America: Honoraria|MedImmune, Allergan, Pfizer, Advanced Liquid Logics, Theravance, Novartis, Merck; Medical Biosurfaces; Locus; Affinergy; Contrafect; Karius;: Grant/Research Support|Novartis, Debiopharm, Genentech, Achaogen, Affinium, Medicines Co., MedImmune, Bayer, Basilea, Affinergy, Janssen, Contrafect, Regeneron, Destiny,: Advisor/Consultant|Sepsis diagnostic: Patent pending|UpToDate: Royalties|Valanbio and ArcBio: Stock Options **Joshua T. Thaden, MD, PhD**, Resonantia Diagnostics, Inc: Advisor/Consultant

